# Reliability of a probabilistic knowledge structure

**DOI:** 10.3758/s13428-024-02468-3

**Published:** 2024-07-25

**Authors:** Debora de Chiusole, Umberto Granziol, Andrea Spoto, Luca Stefanutti

**Affiliations:** 1 FISPPA Department, University of Padua, Padua, Italy; 2 Department of General Psychology, University of Padua, Padua, Italy; 3 Centro di Ateneo Servizi Clinici Universitari Psicologici SCUP, University of Padua, Padua, Italy

**Keywords:** Assessment, Reliability, Classification, Information theory, Latent class analysis, Knowledge structures, Basic local independence model

## Abstract

Indexes for estimating the overall reliability of a test in the framework of knowledge space theory (KST) are proposed and analyzed. First, the possibility of applying in KST the existing classical test theory (CTT) methods, based on the ratio between the true score variance and the total variance of the measure, has been explored. However, these methods are not suitable because in KST error and true score are not independent. Therefore, two new indexes based on the concepts of entropy and conditional entropy are developed. One index is used to estimate the reliability of the response pattern given the knowledge state, while the second one refers to the reliability of the estimated knowledge state of a person. Some theoretical considerations as well as simulations and an empirical example on real data are provided within a study of the behavior of these indexes under a certain number of different conditions.

**Supplementary Information:**

The online version contains supplementary material available at 10.3758/s13428-024-02468-3.

## Introduction

Knowledge space theory (KST; Doignon & Falmagne, 1985, 1999; Falmagne & Doignon, 2010) was developed with the aim of conducting an efficient, accurate and nonnumerical assessment of knowledge. To reach this goal the deterministic and probabilistic components of the theory were developed. Starting from the very basic concept of a *knowledge state*, intended as the set of items in a given *knowledge domain* an individual is able to solve, the deterministic part of KST provided tools and theoretical foundations for the construction and the characterization of different kinds of collections of knowledge states, called *knowledge structures*.

The probabilistic part of the theory was decisive in testing the deterministic models and in the application of KST in the real world for conducting individuals’ knowledge assessment (e.g., ALEKS). The *basic local independence model* (BLIM; Falmagne & Doignon, 1988a) represents the main probabilistic model in KST used to these aims. In particular, the BLIM differentiates between the knowledge state of an individual (which is latent and not directly observable), and its observable counterpart, which is the so-called *response pattern* *R* of an individual. The relation between the response pattern and the knowledge state is defined in the BLIM by three classes of parameters: the probability of each single knowledge state *K* in the population (the parameter $\pi _K$), the *careless error* probability for each item *q* of the knowledge domain (the parameter $\beta _q$), and the *lucky guess* probability for each item (the parameter $\eta _q$). The latter two parameters of an item provide a measure of random error in the two directions of, respectively, a false negative and a false positive. Therefore, they also indirectly provide a measure of reliability of the item. In this respect, it is typical to consider as the reliability of the single item the quantity $1- \beta _q -\eta _q$. Nonetheless, unlike in *classical test theory* (CTT; e.g., Gulliksen, 2013; Novick, 1965) and in *item response theory* (IRT; e.g., Hambleton, Swaminathan, & Rogers, 1991; Lord, 1980; Rasch, 1960), a global reliability index is still missing in KST.

In this article, on the one hand, we show that the classical approach used for the estimation of reliability in CTT cannot be applied within a KST framework. We will show that this is due to different reasons such as the non-independence between the *true score* and the *error*. On the other hand, due to the fact that any reliability index based on the *variance* cannot be used in the KST framework, we propose and test two overall reliability indexes within an information theoretic framework, one for the evaluation of the reliability of a response pattern given a state, and one for the estimation of the reliability of the knowledge state given the observed response pattern.

The paper is structured as follows. In the next section, we introduce some critical issues about the assumptions underlying the traditional CTT reliability indexes. Then we introduce some backgrounds about the estimation of reliability in CTT, about KST and about the evaluation of reliability in this last framework. In Section “The non-independence between the error rate and the “true score”” we show how the crucial issues introduced above impact on the application of traditional reliability indexes in KST. In Section “Reliability of a probabilistic knowledge structure” we introduce the new KST reliability indexes. The following two sections describe, respectively, a simulation study and an empirical example aimed at studying the performance of the new indexes even compared to traditional ones. Finally, in the last section we comment on the main points of the article and discuss some possible future developments in this field.

### Rationale

In classical test theory (CTT; e.g., Gulliksen, 2013; Novick, 1965) and in *item response theory*, the notion of “true score” is central. In the definition provided by Lord and Novick 1968, the *true score* $\tau _{ga}$ of a person *a* on measurement (test) *g* is the expected value of the *observed score* $X_{ga}$. This last is a random variable whose realizations are integer numbers in the set $\{0,1,\ldots ,n\}$, where *n* is the total number of dichotomous items in a test. Thus, one has $\tau _{ga} = \mathcal {E}(X_{ga}),$ where $\mathcal {E}$ denotes expectation. The discrepancy between the observed score and the true score is the *error of measurement* $E_{ga}$, which is the random variable defined by:

$$ E_{ga} = X_{ga} - \tau _{ga}. $$

It is then easily shown that the expectation of the measurement error must be $\mathcal {E}(E_{ga})=0$, irrespective of the value of the true score. In a whole population of individuals, the true score $\tau _{ga}$ is a realization of a “true score” random variable $T_g$, which is defined at a population level. Also random error and observed score can be defined at a population level. They are denoted $E_g$ and $X_g$, respectively, and are regarded as population averages of $E_{ga}$ and $X_{ga}$. Then, it can be shown that the linear model

$$ X_g = T_g + E_g $$

holds true, which is the well-known formula of the CTT. It can also be shown that $T_g$ and $E_g$ are independent random variables. This fact turns out to be fundamental for defining the reliability $\rho _g$ of a test as the ratio between the variance $var(T_g)$ of the true score and the variance $var(X_g)$ of the observed score:

1 $$\begin{aligned} \rho _g = \frac{var(T_g)}{var(X_g)}. \end{aligned}$$

Given the claimed independence, one in fact has $var(X_g) = var(T_g)+var(E_g)$, and hence $\rho _g$ varies between 0 (the observed score only reflects error) and 1 (the observed score equals the true score).

As long as the observed score is a random variable, and as long as the expected value is a property of random variables, in CTT the true score is a property of the observed score and, as such it is neither a primitive concept of the theory, nor a latent construct. A similar status has the measurement error $E_{ga}$. In this sense, it is meaningless, in CTT, to consider the true score in isolation, and dissociated from the expectation of the observed score. This, however, may have its own disadvantages.

Consider the practical situation in which an attainment test *g* (say, in “Algebra”, or any other relevant topic that can be studied at school) consists of $n=10$ multiple choice items, each of which has four response alternatives, with exactly one correct option. Suppose the attainment test is administered to a student *a* who is novel to Algebra and knows literally nothing of the topic. If such a student responds to each of the items at random (e.g., by tossing two regular coins, or by any other relevant method) then, in the long run, the mean of the observed score will approach $.25 \times 10 = 2.5$, because this is the expectation with $n=10$ items and a probability of 1/4 of a lucky guess. Thus, in CTT, $\tau _{ga}=2.5$ is the “true score” of this “totally ignorant” student. This result is somehow counter-intuitive, at least as long as one expects that “total ignorance” should be numerically represented by the least value belonging to the set of all possible “true scores”. The observed score is the number of correct responses and, as such, it is measured on an absolute scale having the 0 as the least value. Therefore, if the “observed score” is meant to be “true score *plus or minus* error” then, once any source of error has been removed (guessing, in this case), the observed score should equal the true score, which is zero (not 2.5) correct answers.

Since the observed score $X_{ga}$ has clear upper and lower bounds (0 and *n*), and since $\tau _{ga}$ is the expectation of such a bounded random variable, it must be bounded too, with the 0 as the lower bound. On the one side, intuition suggests that the “true score” of a totally ignorant student should be 0. On the other side, CTT assumptions lead to the conclusion that such a true score must be greater than zero. A symmetric conclusion would result from considering a student who knows everything in Algebra, but fails some of the items because of inattention or distraction.

This assumption of an identity between a “true score” and the expected observed score is inherent to CTT, but it is not found in other theoretical approaches like, for instance, item response theory (Hambleton et al., 1991; Lord, 1980; Rasch, 1960) or knowledge space theory (Doignon & Falmagne, 1985, 1999; Falmagne & Doignon, 2010), where the two notions are distinct. In fact, both IRT and KST are latent variable models, whereas CTT is not. In those approaches, one thing is the “true score” or the “true state” of an individual, and another thing is the average performance, under standard conditions, of that same individual. The existence of such two different interpretations of what the true score should be, was already clear to Lord and Novik 1968. They named the *operational view* the former, and the *Platonic view* the latter. They were right in recognizing that “true score = expectation” (operational view) is just one of a number of different assumptions. Another one could be, for instance, the median of the observed score, or even its mode. The point is: All these alternative assumptions seem to have the status of arbitrary choices, if no attempt is made of testing them empirically.

If the operational view is false in a given application, then the true score is not necessarily equal to the expected observed score and, hence, the equality

$$ \mathcal {E}(X_{ga}) = \tau _{ga} $$

does not hold anymore. This fact has critical consequences on the definition of “reliability”, a key concept of CTT. First of all, independence between the error term $E_g$ and the true score $T_g$ can no longer be guaranteed. Let $\mathcal {E}(E_g|T_g)$ denote the conditional expectation of the error term, given the true score. Under the operational view, it can be shown to be zero and constant across all possible values of the true score. Under the Platonic view, this may not be the case. It is rather obvious that $\mathcal {E}(E_g|T_g=0)$ and $\mathcal {E}(E_g|T_g=n)$ cannot be the same. In particular, the former will be non-negative and the latter will be non-positive. This argument sufficies to conclude that the error term is not independent of the true score and that, in general, the two will be negatively correlated.

The non-independence between the error term $E_g$ and the true score $T_g$ falsifies the equality $var(T_g + E_g) = var(T_g) + var(E_g)$. In particular, the reliability index would turn out to be

2 $$\begin{aligned} \rho '_g = \frac{var(T_g)}{var(X_g)} = \frac{var(T_g)}{var(T_g)+var(E_g)+2cov(T_g,E_g)}. \end{aligned}$$

This index is still non-negative; however it has no clear upper bound and, with a negative covariance between $E_g$ and $T_g$ it may be greater than 1.

## Background

### Reliability in classical test theory

As described above, the concept of reliability is central in CTT. Therefore, it has been studied from several perspectives and many different methods and indexes have been developed for its assessment. In general, whatever the perspective under which reliability is considered, the problem is that a direct estimation of this property cannot be conducted since the information about the variance of the true score is unknown. Therefore, reliability can only be indirectly estimated. Although none of such indirect measures and indicators are formally linked to the definition of reliability provided above, the way in which such indirect estimation is conducted depends on the particular measurement perspective of the specific test.

In practice, there are three main methods used for estimating the reliability of a test (Lord & Novick, 1968). All of them make use of some assumptions about the equivalence of two tests or two measurements. The first one is the *test–retest method* in which equality between the two measures, up to random error, is assumed, and the correlation between them is considered to be an estimate of the reliability of the test.

The second one is the *parallel forms method* (Brown, 1910; Spearman, 1910), in which all test items of the parallel forms are assumed (i) to measure a single latent variable (unidimensionality), (ii) to use the same scale, (iii) to have the same degree of precision, and (iv) to be subject to the same amount of error. The correlation between the two parallel forms of the test is, in this case, used as an estimation of the reliability of the measure.

The last method takes into account the variance and covariance of the items of a test. The oldest way to conduct such an estimation is the well-known *split-half method* (Brown, 1910; Spearman, 1910), which has been later generalized to the case of *n* parallel parts of a test. When the parallel parts are assumed to be *essentially* $\tau -$*equivalent* each item of the test is assumed to measure the same latent variable on the same scale and different error variances of the items are allowed. Moreover, essential $\tau$-equivalence allows for different degrees of precision of each item. This implies that, under this assumption, each item true score differs by an additive constant (Miller, 1995; Raykov, 1997). This affects items’ true scores means that represent the precision of the items. In this sense, a precise measure is the one that has the values for different items closely grouped. Under all these assumptions, all the items are assumed to have the same factor loading on the latent trait, where a factor loading is the correlation between an item and a theoretical construct that is usually defined as latent trait (or factor).

Under essential $\tau$-equivalence, reliability can be estimated using the most widely applied method, namely *Cronbach’s* $\alpha$ *coefficient* (Cronbach, 1951). Many shortcomings of this coefficient have been highlighted in the literature over the years (e.g., Sijtsma, 2009). Among these, we recall that: (i) when $\tau -$equivalence is not met, $\alpha$ is the lower bound of the reliability of the test (e.g., Sijtsma, 2009; ii) its value is affected by the number of items in the test and the sample size (e.g., Shevlin, Miles, Davies, & Walker, 2000; iii) an extremely high value of the coefficient may not merely reflect reliability, but a certain redundancy in content of the items (e.g., all items are the exact copy of the first item) (e.g., Boyle, 1991); and (iv) the value of $\alpha$ alone is not sufficient for evaluating the reliability of a test (e.g., Schrepp, 2020). To integrate the information obtained through Cronbach’s $\alpha$ coefficient, the *item-rest correlation* should be used as well. Moreover, another evaluation that should be conducted to test in a more accurate way the reliability of a measure is the *pairwise correlation* between the items (Schrepp, 2020).

In general, the assumptions made for the computation of $\alpha$ have been shown to be too restrictive and often unrealistic in the psychological context. More specifically, the assumption of $\tau$-equivalence is particularly critical, and it has been shown that $\alpha$ is not robust to the violation of such assumption (Miller, 1995; Raykov, 1997). Therefore, some different indexes are growing their importance in the estimation of reliability. Among all, *McDonald* $\omega$ (McDonald, 1999) index is the most widely used. It is a function of the factor loadings $\lambda _q$ of the items obtained through confirmatory factor analysis with a single factor. *McDonald* $\omega$ is based on a latent variable model, in contrast to Cronbach’s $\alpha$. Compared to $\alpha$, the main advantages of $\omega$ are: (i) it does not assume $\tau$-equivalence; (ii) it is robust to violations of unidimensionality (see, e.g., Dunn, Baguley, & Brunsden, 2014).

### Knowledge space theory

KST is a nonnumerical approach to the assessment of knowledge. The knowledge of an individual is represented by the *knowledge state* *K*, which is the subset of questions of a specific *knowledge domain* *Q* that she proved to master. The sets *K* and *Q* are not used to produce a quantification of the knowledge of the individual, but they are used to establish what the student is, and what she is not yet able to do. The collection of knowledge states in a population is the so-called *knowledge structure* $\mathcal {K}$.

While the first developments of KST referred especially to its deterministic components, the probabilistic parts of KST were formalized later on by Falmagne and Doignon (1988a, 1988b) in some probabilistic models, the most popular being the restricted latent class model called the basic local independence model (BLIM; Falmagne & Doignon, 1988a). The knowledge state *K* characterizing a given subject is latent and not directly observable, having its observable counterpart in the *response pattern* $R \subseteq 2^Q$. According to the BLIM, the probability *P*(*R*) of a given response pattern $R \subseteq Q$ is obtained as follows:

$$ P(R)= \sum _{K\in \mathcal {K}}P(R|K)\pi _K, $$

where *P*(*R*|*K*) is the conditional probability of the pattern given a state $K\in \mathcal {K}$, and $\pi _K$ is the probability of *K*. In the BLIM, the answers to the items are locally independent given the knowledge state of the person. The conditional probability *P*(*R*|*K*) is determined by two error probability parameters of each item $q\in Q$. Such error parameters are, respectively, the *careless error* $\beta _q$ and the *lucky guess* $\eta _q$, in the interval [0, 1), with $\eta _q+\beta _q <1$. The conditional probability of the pattern *R* given the state *K* depends on the error parameters in the specific sense represented in Eq. 3:

3 $$\begin{aligned} P(R|K)= &  \left( \prod _{q\in K\setminus R} \beta _q\right) \left( \prod _{q \in K \cap R } (1-\beta _q) \right) \nonumber \\\times &  \left( \prod _{q \in R \setminus K} \eta _q \right) \left( \prod _{q \in Q \setminus (K \cup R)} (1-\eta _q) \right) . \end{aligned}$$

The BLIM is, so far, a widely studied model. In fact, several variants of the BLIM have been developed in the years accounting for different measurement conditions and assumptions (Anselmi, Stefanutti, de Chiusole, & Robusto, 2017; de Chiusole, Stefanutti, Anselmi, & Robusto, 2015; Stefanutti, de Chiusole, Gondan, & Maurer, 2020), and even the case of polytomous items through the polytomous local independence model (PoLIM; Stefanutti, de Chiusole, Anselmi, & Spoto, 2020). Moreover, some critical characteristics of BLIM identifiability have been studied in a number of research papers (Heller, 2017; Spoto, Stefanutti, & Vidotto, 2012, 2013; Stefanutti, Heller, Anselmi, & Robusto, 2012; Stefanutti, Spoto, & Vidotto, 2018; Stefanutti & Spoto, 2020).

As reported above, the study of reliability in KST lacks a global index able to provide information about this aspect. In KST literature and practice, some measures are used to, indirectly, estimate the reliability of single items. These are the estimates of $\eta$ and $\beta$ error parameters for each item $q \in Q$ while fitting the BLIM. In general, low values of these parameters are interpreted as an indicator of good reliability of each item. More in detail, it is expected that both parameters are less than .5, and, in any case, that $\eta _q + \beta _q <1$ for all the items.

Reliability in KST is evaluated also with respect to the adaptive assessment conducted to estimate the knowledge state of an individual. In KST, adaptive assessment procedures are computerized algorithms aimed at recovering the state of an individual with the smallest possible number of questions. Typically, in an adaptive assessment procedure, the choice of the next question to ask depends on the responses given to the previous questions. Adaptive assessments are frequently used to evaluate the knowledge of a person on a certain subject (Falmagne, Albert, Doble, Eppstein, & Hu, 2013), or to assess the most exhaustive set of symptoms a person may present in case of mental disorders (Spoto, Stefanutti, & Vidotto, 2010).

Considering the application of adaptive assessment in the field of education, all the main methods used to estimate assessment reliability are based on the so-called *extra question procedure* (Falmagne et al., 2013). A question *p*, which is not linked to the ongoing adaptive procedure, is randomly selected in a uniform distribution on the set of all problems *Q* and administered to the participant. The answer to *p* is then compared with (i) the presence/absence of *p* in the final estimated knowledge state of the individual; or (ii) the probability of a correct/wrong answer to *p* calculated at several points during the adaptive procedure. In the former case, a $2 \times 2$ matrix is built computing, for all participants, the correct/wrong answer to the extra question, and its presence/absence in the final state. Indicators like tetrachoric or $\phi$ correlation coefficients can be then computed to evaluate the reliability of the assessment. The latter case consists in the computation of the correlation between the actual (correct/wrong) answer to the extra question *p* and the estimation of the probability of a correct answer to *p*. Such estimation can be conducted a number of times along the assessment. The number of probability evaluations and their location along the assessment path are established through the application of the Vincent curve analysis method (Vincent, 1912). This method consists in splitting different assessments in the same number of parts, identifying one probabilistic knowledge state per part, and computing the point biserial correlation between the estimated probability of solving the extra question in all the selected states and the actual answer to the extra question (Falmagne et al., 2013).

## The non-independence between the error rate and the “true score”

In classical test theory (Gulliksen, 2013; Novick, 1965), the reliability index relies on the independence between true score and error. Although a notion like the “true score” is not considered or developed in KST, it is always possible to define one in a rather easy and natural way, as long as the items are dichotomous. If $K \in \mathcal {K}$ is the “true knowledge state” of an individual, then the cardinality |*K*| can be retained as the “true score” of that individual. In an analogous way, the cardinality |*R*| of the observed response pattern $R \in 2^Q$ is the “observed score”. With these definitions, every individual has a “true score” |*K*| and an observed score |*R*|. Thus, with |*Q*| items, both the true and the observed scores belong to the set $V = \{0,1,\ldots ,|Q|\}$.

Like in CTT, within the same individual, the true score $t = |K|$ is assumed to be constant, whereas the observed score is a random variable, whose realizations vary from one testing occasion to another. Let *X* represent such a random variable. The realizations of *X* are the numbers in *V*. If $x_i \in V$ is the realization of *X* in testing occasion *i*, then $\epsilon _i = x_i - t$ is the error term. Thus the error term is, itself, a random variable whose realizations belong to $\{x-t: (x,t) \in V \times V\}$. Let *E* denote such a random variable. The third random variable is the true score *T*, which varies across individuals, and has realizations in *V*. Then, the error term *E* is independent of the true score *T* if and only if the conditional expectation $\mathcal {E}(E|T)$ is constant across all possible realizations of the true score *T*. We show that in KST this cannot happen, in general.

For $q \in Q$, let $X_q$ be a random variable, whose realizations are in $\{0,1\}$, which represents the observed response (1 = correct, 0 = incorrect) to item *q*. In the BLIM, the conditional expectation of $X_q$, given knowledge state $K \in \mathcal {K}$ is

$$\begin{aligned} \mathcal {E}(X_q|K) = 1 \times P(X_q = 1|K) + 0 \times P(X_q=0|K) = P(X_q=1|K), \end{aligned}$$

hence

$$ \mathcal {E}(X_q|K) = {\left\{ \begin{array}{ll} 1-\beta _q & \text {if}\, q \in K \\ \eta _q & \text {if}\, q \in Q \setminus K. \end{array}\right. } $$

From this, one can easily obtain the conditional expectation of the observed score, which turns out to be

$$ \mathcal {E}(X|K) = \sum _{q \in Q}\left( (1-\beta _q)I(q,K) + \eta _q(1-I(q,K))\right) , $$

where *I*(*q*, *K*) is the set membership index (i.e., $I(q,K)=1$ if $q \in K$ and $I(q,K)=0$ otherwise). This gives

4 $$\begin{aligned} \mathcal {E}(X|K) = |K| - \sum _{q \in K} \beta _q + \sum _{q \in Q \setminus K} \eta _q. \end{aligned}$$

On the other side, given any “true score” $t \in V$, the conditional expectation of the observed score *X*, given the true score *t* is

$$ \mathcal {E}(X|t) = \sum _{x \in V} x P(X=x|t). $$

This last can be rewritten as

5 $$\begin{aligned} \mathcal {E}(X|t)&= \sum _{x \in V} x \sum _{K \in \mathcal {K}} P(X=x|K)P(K|t) \nonumber \\&= \sum _{K \in \mathcal {K}} P(K|t)\sum _{x \in V} x P(X=x|K) \nonumber \\&= \sum _{K \in \mathcal {K}} P(K|t)\mathcal {E}(X|K), \end{aligned}$$

where, for $$\mathcal {K}_t = \{K \in \mathcal {K}: |K| = t\}$$,

$$ P(K|t) = \frac{\pi _K}{\sum _{K \in \mathcal {K}_t} \pi _K} $$

is the conditional probability of knowledge state $K \in \mathcal {K}_t$ given true score *t*, and $P(K|t)=0$ for $K \notin \mathcal {K}_t$. Now, plugging Eq. 4 into Eq. 5, and after some algebra, one obtains

$$\begin{aligned} \mathcal {E}(X|t)&= \frac{\sum _{K \in \mathcal {K}_t}\left( |K| - \sum _{q \in K} \beta _q + \sum _{q \in Q \setminus K} \eta _q \right) \pi _K}{\sum _{K \in \mathcal {K}_t} \pi _K}\\&= t + \frac{\sum _{K \in \mathcal {K}_t}\left( \sum _{q \in Q \setminus K} \eta _q - \sum _{q \in K} \beta _q\right) \pi _K}{\sum _{K \in \mathcal {K}_t} \pi _K}. \end{aligned}$$

It follows at once that the conditional expectation of the error term is

$$ \mathcal {E}(E|t) = \mathcal {E}(X|t) - t = \frac{\sum _{K \in \mathcal {K}_t}\left( \sum _{q \in Q \setminus K} \eta _q - \sum _{q \in K} \beta _q\right) \pi _K}{\sum _{K \in \mathcal {K}_t} \pi _K}, $$

which, in general, is different from zero, and varies with the true score *t*.

### Example 1: True score $T_g$ and error $E_g$ are not independent

This example is aimed at comparing the true score $T_g$ of a subject with her observed score $X_g$. In particular, it is shown that the error term $E_g$ is not independent from the true score $T_g$, as discussed in the previous section. To this aim, first some data sets were simulated under a particular “true” score, then the distributions of the observed scores were obtained. A detailed description of this process follows.

The cardinality of the domain *Q* of a fictitious test was fixed at 10 items. Two scenarios were considered that vary in the dimensionality assumed for the measured latent variable. In the former scenario, a unidimensional variable was assumed, represented by the knowledge structures $\mathcal {K}_{l}$ derived assuming a linear order among the items. Indeed, in a traditional psychological assessment framework the computation of a reliability index only makes sense if the scale is unidimensional. In the latter scenario, multidimensionality was considered, represented by the structure $\mathcal {K}_{rand}$ generated at random, whose cardinality was equal to 100. More in detail, it was obtained by computing $\{\emptyset ,Q\} \cup \mathcal {L}$, where $\mathcal {L}$ was generated at random, using sampling without replacement on the power set $2^Q$.

In both scenarios, the manipulated variables were the probability distributions on the states, and the “type” and the “amount” of error in the data, via $\beta _q$ and $\eta _q$ parameters of the BLIM. Concerning the probability distribution, two conditions were considered. In the former, the uniform distribution was used on the set of states. In the latter, a non-uniform distribution was used. This last was obtained by generating a number of uniformly distributed random values in the interval (0, 1) equal to the number of knowledge states. Such random numbers were subsequently normalized to sum up to 1.

**Fig. 1:**
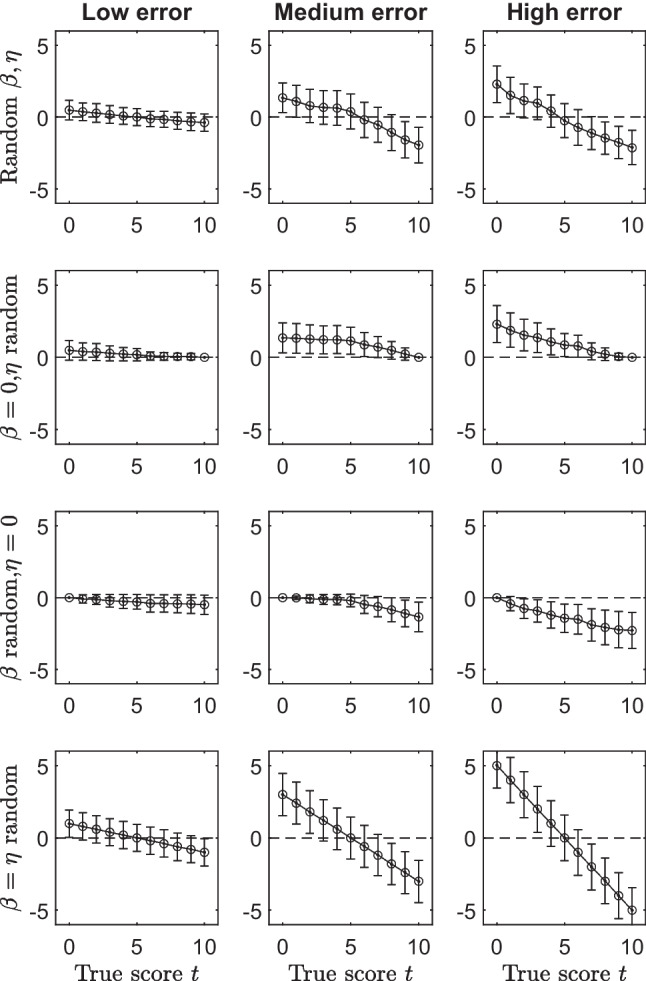
Error term $\bar{\Delta }_t$ as a function of the true score $t \in \{0,1,\cdots ,10\}$. *Error bars* are standard deviations. See text for more details

Concerning the type and the amount of error, 3 amounts $\times$ 4 types $=12$ conditions were considered. The “amount” of error was manipulated through the upper bound *a* of the interval used for generating the error parameters, which was chosen among .10, .30, or .50. These values represent a situation of low, medium, and high error in the data. The four types of error were (for each $q \in Q$):

- $\beta _q,\eta _q >0$ generated from a uniform distribution in the interval (0, *a*];
- $\beta _q=0$ and $\eta _q>0$ generated from a uniform distribution in the interval (0, *a*];
- $\eta =0$ and $\beta _q>0$ generated from a uniform distribution in the interval (0, *a*];
- $\beta _q=\eta _q>0$ generated from a uniform distribution in the interval (0, *a*].

These types of error represent, respectively, the situation in which the observed score can be either smaller or greater than the true score; the situation in which the observed score can only be greater than the true score; the situation in which the observed score can only be smaller than the true score; and the situation in which the observed score can be either smaller or greater than the true score, where overestimations and the underestimations are equally likely for each item. The parameters were kept equal across the two scenarios.

In the former scenario, for each state $K_t \in \mathcal {K}_{l}$, with $t \in \{0,1,\cdots ,10\}$, a number of 10,000 response patterns $R_{tj}$ ($j \in \{1,2,\cdots ,10^4\}$) were generated in each error condition. More precisely, for every item $q \in Q$, each response pattern $R_{tj}$ was obtained by introducing in $K_t$ false negatives and false positives with probabilities $\beta _q$ and $\eta _q$, respectively. Thus, a number of $11 \times 10,000=110,000$ pairs $(K_t,R_{tj})$ were generated. For each pair $(K_t,R_{tj})$, the observed and true scores were computed, respectively, as the cardinalities $|R_{tj}|$ and $|K_t|$. Then, the discrepancy $\Delta _{tj}=|R_{tj}|-|K_t|$ was obtained. It is worth noticing that the discrepancy $\Delta _{tj}$ can be considered as a realization of the error term $E_g$. For each true score *t*, the average value $\bar{\Delta }_{t}=10^{-4} \sum _{j=1}^{10^{4}}\Delta _{tj}$ and the corresponding standard deviation were computed. The same procedure was used in the latter scenario.

Figure 1 displays the results obtained for the first scenario where $\mathcal {K}_{l}$ was used for generating the data, and a uniform distribution was assumed across the states of the structure. Row panels represent the four considered types of error, whereas column panels represent the three amounts of error. In each panel, the true score *t* is along the *x*-axis, whereas the error term $\bar{\Delta }_{t}$ is along the *y*-axis. Moreover, bullet points represent the average values $\bar{\Delta }_{t}$, error bars refer to their standard deviations, and circles indicate the theoretically expected amount of error $\epsilon$.

It is rather evident from the figure that the error term $\bar{\Delta }_{t}$ is monotonically decreasing in the true score *t*, irrespective of the type and the amount of error used for generating the data. Thus, the true score *t* and $\bar{\Delta }_{t}$ are not independent. Moreover, the expected amount of error $\epsilon$ (circles in each panel) always coincides with the average value $\bar{\Delta }_{t}$.

What changes among error conditions due to the manipulation of the amount of error (columns in the figure) is the range $|\bar{\Delta }_{10}-\bar{\Delta }_{0}|$ that widens, as expected, when the error increases.

The effect of the type of error is less trivial. When $\beta _q=0$ (second row of the figure), values of $\bar{\Delta }_{t}$ are always positive or equal to zero. In this condition, the difference between the observed and the true scores only depends on false positives (i.e., $\eta _q$), meaning that the observed score is always greater than or equal to the true one. Thus, the greater the true score the smaller the probability of observing false negatives. Moreover, this probability is equal to zero when $t=10$ (maximum score.) When $\eta _q=0$ (third row of the figure), values of $\bar{\Delta }_{t}$ are always negative or equal to zero. In this condition, the difference between the observed and the true scores only depends on false negatives (i.e., $\beta _q$), meaning that the observed score is always smaller than or equal to the true one. Thus, the greater the true score the greater the probability of observing false negatives. Moreover, this probability is equal to zero when $t=0$ (minimum score.) When $\beta _q,\eta _q >0$ (first and last row of the figure), the effect of false positive and false negative is counterbalanced, with a smaller probability of the false positives and a greater probability of the false negatives as the true score increases. When $\beta _q=\eta _q$ for all items $q \in Q$(last row of the figure), this counterbalancing is perfect, with $\bar{\Delta }_{t}=0$ exactly when the true score is $t=5$.

Similar results were obtained in the other scenario of the study (see supplementary material of the article). Thus, we observe in passing that there are no appreciable differences between the unidimensional and the multidimensional scenarios.

### Example 2: The CTT-based reliability index exceeds 1

As argued in the previous section, if $T_g$ and $E_g$ are independent random variables, $\rho _g$ varies between 0 (the observed score only reflects error) and 1 (the observed score equals the true score). The aim of this section is to show what happens to the range of the reliability $\rho _g$ when $T_g$ and $E_g$ are not independent.

To this aim, the CTT-based reliability index was computed as in Eq. 1 under different conditions of error and by using different types of probability distribution on the states. A detailed description of the procedure follows.

As in the Example described in Section “[Sec Sec7]”, the cardinality of the domain *Q* was fixed to 10 items and the knowledge structure $\mathcal {K}_l$ was derived assuming a linear order among the items. The manipulated variables were (i) the amount of error in the data, via $\beta _q$ and $\eta _q$ parameters of the BLIM, and (ii) the probability distribution $\pi _{\mathcal {K}_l}$ on the states in $\mathcal {K}_l$. Three error conditions were considered. The interval used for generating the $\beta _q$ and $\eta _q$ parameters of each item $q \in Q$ were (0.10], (0.30], and (0.50], respectively in the low, medium, and high error conditions. The probability distribution $\pi _{\mathcal {K}_l}$ was manipulated under three different conditions. What varied among these three conditions was the fact that $\pi _{\mathcal {K}_l}$ could be more or less close to a uniform distribution. More in detail, these three probability distributions were drawn from a $|\mathcal {K}_l|$-dimensional Dirichlet distribution, with parameters $\alpha _{K_1},\ldots ,\alpha _{K_{11}}=\alpha$ and $\alpha \in \{1,5,30\}$. When all $\alpha _{K}$ parameters are equal to the constant $\alpha$, the Dirichlet distribution is named ‘symmetric’. In particular, when $\alpha = 1$, the symmetric Dirichlet distribution is uniform over the entire simplex of all the $|\mathcal {K}_l|$-dimensional points, each of which is a different probability distribution on the set $\mathcal {K}_l$ of knowledge states. On the other hand, when $\alpha$ tends to $+\infty$, the mass of the symmetric Dirichlet distribution is almost all concentrated within a neighborhood of the centroid of the simplex, which coincides with the uniform distribution over $\mathcal {K}_l$. This implies that, as the $\alpha$ parameter of the symmetric Dirichlet distribution tends to $+\infty$, the expected Euclidean distance between the randomly sampled distribution $\pi _{\mathcal {K}_l}$ and the uniform distribution over $\mathcal {K}_l$ tends to zero.

In each of the $3 \times 3=9$ conditions, a number of 1,000 samples, each of size 1,000 were generated under the BLIM. Then, for each sample of each condition, the true score was computed as the cardinality of the estimated *K*, and the observed score was computed as the cardinality of *R*. Finally, in each sample, the CTT-based reliability index was computed as in Eq. 1.

**Fig. 2:**
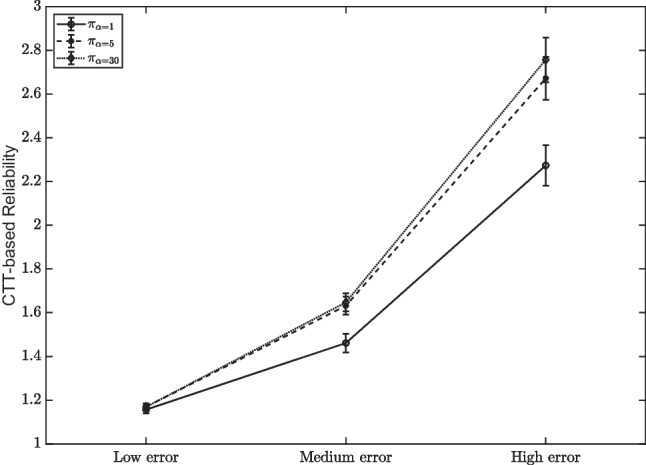
CTT-based reliability index computed in Section “Example 2: The CTT-based reliability index exceeds 1”. The three error conditions are along the *x*-axis, the estimated CTT-reliability index is along the *y*-axis, and the three curves refer to probability distributions. The *straight line* represents the “strongly non-uniform” distribution, the *dotted line* represents the “quasi-uniform” distribution, and the dashed line represent an indeterminate situation

Figure 2 shows the results. Error conditions are along the *x*-axis, the estimated CTT-reliability index is along the *y*-axis, and the three curves refer to probability distributions: the straight line represents a strong departure from a uniform distribution, the dashed line represents a moderate departure from the uniform distribution, and the dotted line represents a small departure from the uniform distribution.

Some interesting results follow. First, the CTT-based reliability exceeds one, irrespective of the amount of error and the probability distribution used for generating the data. Moreover, it increases as the probability distribution is close to the uniform one, and as the amount of error in the data increases.

This result follows from two observations. First, if the distribution on the knowledge states is fixed, then the variance of the true score is fixed too, and hence the numerator of Eq. 2 is a constant and does not depend on the amount of error. On the other side, by increasing the error, the covariance between true score and error becomes more negative, thus decreasing the denominator of Eq. 2. The result is an increased value of the CTT-based reliability index. Second, if the error is fixed, and the distribution on the knowledge states varies in a way that increases error variance, then the numerator increases, and so does the value of the CTT-based reliability index. Both these results are a mere effect of non-independence between the true score and the error.

## Reliability of a probabilistic knowledge structure

Unlike CTT and IRT, a reliability index seems to be missing in KST. The notion of reliability in CTT relies upon a signal-to-noise ratio principle (henceforth referred to as the S/N principle). The reliability is the ratio of the true score variance to the true score variance plus error variance. It basically says how much of the observed variance is accounted for by the true score variance.

In KST, the role of the true score *T* is played by the “knowledge state” $\textbf{K}$, a random variable whose realizations are the knowledge states in $\mathcal {K}$. Similarly, the role of the observed score *X* is played by the observed response pattern $\textbf{R}$, a random variable whose realizations are in the power set $2^Q$. Since both $\textbf{K}$ and $\textbf{R}$ are categorical random variables, the variance is not a property of their probability distributions. Nonetheless, by following the same S/N principle as that in CTT, it is possible to derive a reliability measure for the two random variables $\textbf{K}$ and $\textbf{R}$, within an information theoretic framework.

### Information theoretic measures

By replacing variance with entropy, reliability is here re-defined as the amount of information needed for describing the observed response pattern that is accounted for by the true knowledge state. The amount of information in the response pattern that is not accounted for by the knowledge state is regarded as “noise” or, simply, “error”. In information theory, such type of noise is measured by Shannon’s entropy (Shannon, 1948) which, for a discrete random variable *X*, is defined as

$$ H(X)=-\sum _{x \in \mathcal {X}} P(x)\log P(x), $$

where $\mathcal {X}$ denotes the support of *X*, and *P*(*x*) is the probability to observe the realization *x*. If $\log$ is the base 2 logarithm, then *H*(*X*) is measured in bits of information.

Particularly relevant here is the notion of “conditional entropy” of a random variable, given another random variable (Cover, 1999). Given two random variables *X* and *Y*, the *conditional entropy* *H*(*Y*|*X*) of *Y*, given *X* is a measure of the expected amount of information that is needed to describe the outcome of *Y*, given that the outcome of *X* is known. Such amount is zero if the outcome of *Y* is completely determined by that of *X*, and it equals the entropy *H*(*Y*) of the random variable *Y* if *X* and *Y* are independent random variables. Thus, the double inequality

6 $$\begin{aligned} 0 \le H(Y|X) \le H(Y) \end{aligned}$$

holds true in general. Denoting by $\mathcal {X}$ and $\mathcal {Y}$ the supports for *X* and *Y* respectively, *H*(*Y*|*X*) is defined as

$$ H(Y|X)= -\sum _{x \in \mathcal {X}}\sum _{y \in \mathcal {Y}} P(x,y)\log \frac{P(x,y)}{P(x)}. $$

Closely related to the conditional entropy is the *mutual information* between two random variables. It is defined as the difference between the (unconditional) entropy of *Y* and its conditional entropy given *X*:

$$ I(X,Y) = H(Y)-H(Y|X). $$

The mutual information is symmetric (i.e., $I(X,Y)=I(Y,X)$) and satisfies the double inequality:

$$ 0 \le I(X,Y) \le \min \{H(X),H(Y)\}. $$

Finally, a standardized measure, derived from the mutual information is the *uncertainty coefficient*. It is obtained as the ratio of the mutual information to the entropy of *Y*:

$$ U(Y|X) = \frac{I(X,Y)}{H(Y)} = 1-\frac{H(Y|X)}{H(Y)}. $$

Such coefficient is asymmetric (i.e., $U(X|Y)\ne U(Y|X)$) and such that $0 \le U(Y|X) \le 1$. Of the total amount of bits that are needed for describing *Y*, the uncertainty coefficient *U*(*Y*|*X*) provides the proportion accounted for by the random variable *X*. For this reason, its application to a probabilistic knowledge structure can be regarded as a reliability coefficient.

### Derivation of a reliability coefficient

The fundamental equation that relates the two random variables $\textbf{R}$ and $\textbf{K}$ is that of a probabilistic knowledge structure, that is, for $R \in 2^Q$ and $K \in \mathcal {K}$,

$$ P(\textbf{R}=R) = \sum _{K \in \mathcal {K}}P(\textbf{R}=R|\textbf{K}=K)P(\textbf{K}=K). $$

To lighten the notation, let $P(R) = P(\textbf{R}=R)$, $\pi _K = P(\textbf{K}=K)$, and $P(R|K)=P(\textbf{R}=R|\textbf{K}=K)$. Then the Shannon entropies for the two random variables $\textbf{R}$ and $\textbf{K}$ are

7 $$\begin{aligned} H(\textbf{K}) = -\sum _{K \in \mathcal {K}} \pi _K \log \pi _K, \end{aligned}$$

and

8 $$\begin{aligned} H(\textbf{R}) = -\sum _{R \in 2^Q}\sum _{K \in \mathcal {K}} P(R|K)\pi _K \log \sum _{K \in \mathcal {K}}P(R|K)\pi _K, \end{aligned}$$

whereas, the conditional entropy of $\textbf{R}$ given $\textbf{K}$ is

9 $$\begin{aligned} H(\textbf{R}|\textbf{K}) = -\sum _{R \in 2^Q}\sum _{K \in \mathcal {K}}P(R|K)\pi _K\log P(R|K). \end{aligned}$$

Then, a reliability coefficient is obtained as

10 $$\begin{aligned} U(\textbf{R}|\textbf{K})=1-\frac{H(\textbf{R}|\textbf{K})}{H(\textbf{R})}. \end{aligned}$$

The new index computed by Eq. 10 is named RP-Reliability (i.e., response pattern reliability) index.

Similarly, and even more importantly, we can derive a reliability index for a state *K* given a response pattern *R*, as follows. For the chain rule of conditional entropy,

$$ H(\textbf{R}|\textbf{K}) = H(\textbf{R},\textbf{K}) - H(\textbf{K}) $$

and

$$ H(\textbf{K}|\textbf{R}) = H(\textbf{K},\textbf{R}) - H(\textbf{R}) $$

where $H(\textbf{R},\textbf{K}) = H(\textbf{K},\textbf{R})$ is the joint entropy of *R* and *K*.

It follows that

$$ H(\textbf{K}|\textbf{R}) = H(\textbf{R}|\textbf{K}) + H(\textbf{K}) - H(\textbf{R}) $$

which gives

11 $$\begin{aligned} U(\textbf{K}|\textbf{R})&= 1 - \frac{H(\textbf{K}|\textbf{R})}{H(\textbf{K})} = \frac{H(\textbf{K}) - H(\textbf{R}|\textbf{K}) - H(\textbf{K}) + H(\textbf{R})}{H(\textbf{K})}\nonumber \\&=\frac{H(\textbf{R}) - H(\textbf{R}|\textbf{K})}{H(\textbf{K})} \end{aligned}$$

This index, named KS-Reliability (i.e., knowledge state reliability), reports the amount of information needed for describing *K*, which is accounted for by the pattern *R*. Therefore, it can be conceived as an indicator of the reliability of real assessment.

It is finally observed that, by the inequality in Eq. 6, both $H(\textbf{R}|\textbf{K}) \le H(\textbf{R})$ and $H(\textbf{K}|\textbf{R}) \le H(\textbf{K})$ hold true. Therefore, both $U(\textbf{R}|\textbf{K})$ and $U(\textbf{K}|\textbf{R})$ belong to the interval [0, 1].

In particular, $U(\textbf{R}|\textbf{K}) = 0$ whenever $H(\textbf{R}|\textbf{K})=H(\textbf{R})$ and this holds true if and only if $\textbf{R}$ and $\textbf{K}$ are independent random variables. In total analogy, $U(\textbf{K}|\textbf{R})=0$ if and only if there is stochastic independence between the two random variables. In other words, when $U(\textbf{K}|\textbf{R}) = 0$, knowing the response pattern does not help to infer the knowledge state. Conversely, when $U(\textbf{R}|\textbf{K})=0$, knowing the knowledge state does not help to predict the response pattern. A sufficient condition for $U(\textbf{R}|\textbf{K}) = U(\textbf{K}|\textbf{R}) = 0$ is provided by $\beta _q=\eta _q=1/2$ for all items $q \in Q$. It is easily seen that, in this case, $P(R|K)=2^{-|Q|}$ for all $R \in 2^Q$ and $K \in \mathcal {K}$. Therefore we have

$$ P(R) = \sum _{K \in \mathcal {K}} P(R|K)\pi _K = 2^{-|Q|}\sum _{K \in \mathcal {K}} \pi _K = 2^{-|Q|}, $$

which gives $P(R|K)=P(R)$ for all *R* and all *K*.

On the other side, $U(\textbf{R}|\textbf{K})=1$ whenever error parameters $\beta _q$ and $\eta _q$ are simultaneously zero for all $q \in Q$. This fact can be shown under the convention that $0 \cdot \log 0$ equals zero (because $\lim _{x \downarrow 0} x \log x = 0$). Then we have $U(\textbf{R}|\textbf{K}) = 0$ if and only if $H(\textbf{R}|\textbf{K}) = 0$. This can occur if and only if

$$ \sum _{K \in \mathcal {K}}\sum _{R \in 2^Q} P(R|K)\pi _K \log P(R|K) = 0. $$

If $\beta _q = \eta _q = 0$ for all $q \in Q$ then

$$ P(R|K) = {\left\{ \begin{array}{ll} 0 & \text {if}\, {R \ne K} \\ 1 & \text {if}\, {R = K} \end{array}\right. } $$

and hence, in this case, $H(\textbf{R}|\textbf{K}) = \sum _{K \in \mathcal {K}} \pi _K \log 1 = 0$. On the other side if either $\beta _q > 0$ or $\eta _q > 0$ for some $q \in Q$, then there will be $K \in \mathcal {K}$ and $R \in 2^Q$ with $K \ne R$ and $P(R|K) > 0$, entailing $H(\textbf{R}|\textbf{K}) > 0$. Analogous conclusions can be drawn for $U(\textbf{K}|\textbf{R}) = 1$. Thus, $U(\textbf{R}|\textbf{K})=U(\textbf{K}|\textbf{R})=1$ if and only if all the error parameters are zero, so that there is an identity between the observed response pattern and the latent knowledge state.

A numerical value for each of the two indexes $U(\textbf{K}|\textbf{R})$ and $U(\textbf{R}|\textbf{K})$ could only be obtained if the true values of the $\beta _q$, $\eta _q$, and $\pi _K$ parameters of the BLIM were known. In empirical applications of the BLIM, this is not the case. Instead, one obtains point estimates $\hat{\beta }_q$, $\hat{\eta }_q$, and $\hat{\pi }_K$ of the BLIM parameters (e.g., maximum likelihood estimates) from proper data sets. In that case, estimated values for the two indexes $\hat{U}(\textbf{K}|\textbf{R})$ and $\hat{U}(\textbf{R}|\textbf{K})$ can be obtained by replacing the true, though unknown, values of the BLIM parameters, with their estimates.

## Simulation study: KST-based vs. CTT-based reliability indexes

With the aim of analyzing the behavior of the KST-based reliability indexes and comparing them with Cronbach’s $\alpha$ and McDonald’s $\omega$ CTT-based reliability indexes, a simulation study was carried out. Several variables (e.g., sample size, amount of error in the data, cardinality of knowledge structures) were manipulated in different scenarios that were used for generating the responses to a set of items forming a “fictitious test”. Then, the two KST-based reliability indexes were estimated on the generated samples together with Cronbach’s $\alpha$ and McDonald’s $\omega$ CTT-based reliability indexes.

### Simulation design and data set generation

The cardinality of the domain *Q* of the fictitious test was fixed at ten items. This number was chosen because (a) it is not so small to be unrealistic in practice, and (b) it is not so big to make simulations too time-consuming. The reliability of this fictitious test was estimated on the simulated data sets that were generated under different conditions. Table 1 shows the variables manipulated in the different conditions.

**Table 1:** Design of the simulation study used for generating the data

Cond.	$|\mathcal {K}|$	Item error probabilities	*N*
1	2	(0,.10],(0,.20],(0,.30],(0,.40]	20
2	11	(0,.10],(0,.20],(0,.30],(0,.40]	110
3	200	(0,.10],(0,.20],(0,.30],(0,.40]	2000
4	500	(0,.10],(0,.20],(0,.30],(0,.40]	5000

Column one displays the condition number whereas Columns 2 to 4 display the manipulated variables for generating the data. In particular, Column 2 displays the cardinality of the knowledge structures, Column 3 displays the item error probability intervals, and Column 4 displays the two sample sizes

The data were simulated by using four knowledge structures having different cardinalities (Column 2). Each structure was generated as follows. A structure $\mathcal {K}_i$ (with $i \in \{1,2, 3, 4\}$ representing the condition number) and a probability distribution $\pi _{\mathcal {K}_i}$ on $\mathcal {K}_i$ were assumed. The structure $\mathcal {K}_i$ was obtained by computing $\{\emptyset ,Q\} \cup \mathcal {L}$, where $\mathcal {L}$ was generated at random, using a sampling without replacement on the power set $2^Q\setminus \{\emptyset ,Q\}$. It was assumed that $\pi _{\mathcal {K}_i}$ was the uniform distribution, so that the probability of each state $K \in \mathcal {K}_i$ was equal to $1/|\mathcal {K}_i|$.

Among the generated structures, two of them are of particular interest, that is $\mathcal {K}_1$ and $\mathcal {K}_2$. Structure $\mathcal {K}_1=\{\emptyset ,Q\}$ is composed of the two states representing individuals that answer to all items in the same way. This structure conforms with the desirable CTT situation in which all items measure exactly the same variable. The items of structure $\mathcal {K}_2$ also measure the same latent variable (as it is unidimensional), although at different levels. In fact, structure $\mathcal {K}_2$ is composed of the 11 states that are obtained assuming a total order among the items. This structure represents the situation of a unidimensional test where all items are aligned along a single dimension. The other two structures $\mathcal {K}_3$ and $\mathcal {K}_4$ represent two different multidimensional situations, with the former having fewer states (i.e., 200) than the latter (i.e., 500).

To make sure that in the randomly generated knowledge structures $\mathcal {K}_3$ and $\mathcal {K}_4$ the distances among states are not considerably larger than in the theoretically generated structures $\mathcal {K}_1$ and $\mathcal {K}_2$, an average minimum distance was computed for all of them. First, the minimum distance of every knowledge state $K \in \mathcal {K}$ from all the others was computed. Then, the mean and the standard deviation of all the minimum distances were computed for each structure. The obtained means (standard deviations) were 10 (0), 1 (0), 1.10 (.30), and 1.01 (.09), respectively, for $\mathcal {K}_1$, $\mathcal {K}_2$, $\mathcal {K}_3$, and $\mathcal {K}_4$. It is worth noticing that the average minimum distances of $\mathcal {K}_3$, and $\mathcal {K}_4$ are not appreciably larger than the minimum possible value, which is 1.

Another variable that was manipulated for generating the data is the item error probability, more precisely the false negative $\beta _q$ and the false positive $\eta _q$ probabilities of the BLIM. For each item *q*, these parameters were randomly extracted under the uniform distribution by using the four different intervals displayed in Column 3 of Table 1. Moreover, the no-error condition was also considered.

For generating the simulated response patterns, first, a knowledge state *K* was sampled from structure $\mathcal {K}_i$, with probability $\pi _K$. Then, for every item $q \in Q$, random false negatives and false positives were produced in the response pattern with probabilities $\beta _q$ and $\eta _q$.

For each condition of the study, a sample size was considered that depended on the cardinality of the respective structure (last column of Table 1), that is $10|\mathcal {K}_i|$.

For each of the 4 (structures) $\times 5$ (item error probabilities) $\times 1$ (samples size1) $= 20$ conditions, 100 samples were simulated.

### Methods

The BLIM was fitted to each of the $20 \times 100 =2,000$ generated data sets. The model parameters were estimated by maximum likelihood via the expectation maximization algorithm (Stefanutti & Robusto, 2009) and they were used for computing the KST-based reliability indexes as in Eqs. 10 and 11. Then, in each condition of the study, average values (and the corresponding standard deviations) of both indexes were estimated across the 100 simulated samples. The average estimates obtained for the RP-reliability and the KS-reliability indexes were compared with their theoretical values. These last were obtained by an application of Eqs. 10 and 11 to the “true” values of the BLIM parameters that were used for generating the data.

Moreover, other two indexes were computed that is the Hamming distance and an *accuracy rate* (AR). Both of them compare the *true knowledge state* *K* used for generating a response pattern *R*, with the *modal knowledge state* $\hat{K}$ estimated from *R*. For each response pattern *R* in the sample, the modal knowledge state $\hat{K}$ is the state having the maximum of the conditional probability

$$ P (K | R) =\frac{ P (R | K) \pi (K)}{P(R)}. $$

The Hamming distance $K \Delta \hat{K}$ is then computed by

$$ K \Delta \hat{K}=|(\hat{K} \setminus K) \cup (K \setminus \hat{K})|. $$

This distance is also known as the symmetric distance between two sets. Then, for each subject *s* in sample $\mathcal {D}$ of size *N*, the AR is computed by

$$ AR=\frac{1}{N}|\{s \in \mathcal {D}:K_s \Delta \hat{K}_s=0\}|, $$

that is the proportion of subjects in the sample whose modal knowledge state $\hat{K}$ is equal to the true one *K*.

In each condition of the study, average values (and the corresponding standard deviations) of all these indexes were computed across the 100 simulated samples. It is worth noticing that these two indexes can be interpreted only if the BLIM parameters are identifiable. Thus, the BLIM’s identifiability was checked using the BLIMIT function (Stefanutti et al., 2012) with each of the four knowledge structures used in the simulation design. Knowledge structure $\mathcal {K}_2$, which corresponds to a total order on the set of items, turned out to be the only unidentifiable structure. In particular, the $\eta _q$ parameter of the easiest item and the $\beta _q$ parameter of the most difficult one were unidentifiable. This issue was addressed by setting the estimates of these two parameters equal to their true values. Of course, in an empirical study true values are unknown, and, therefore, a different route should be found (some comments are provided in Section “Discussion and final remarks”).

Finally, Cronbach’s $\alpha$ and McDonald $\omega$ were computed on the data sets.

### Results

**Fig. 3:**
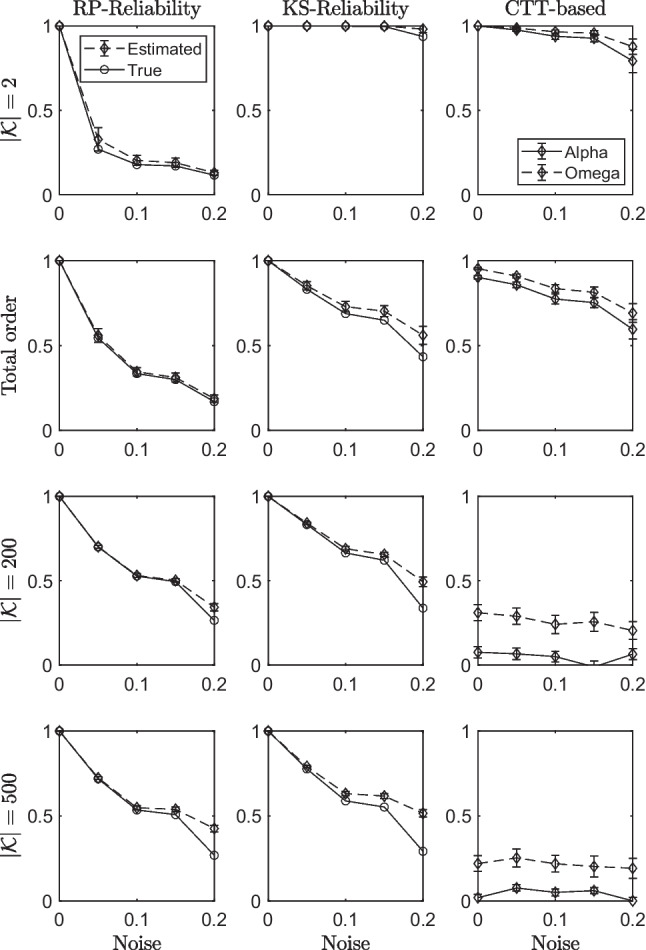
Results obtained in each condition (row-panels of the figure) of the simulation study, for the KST-based reliability indexes (RP-Reliability in Column 1, KS-Reliability in Column 2), and for the Cronbach’s $\alpha$ and McDonald’s $\omega$ indexes (Column 3)

Figure 3 shows the results obtained in each condition of the study (row-panels of the figure) for the KST-based reliability indexes (RP-Reliability in the first column, KS-Reliability in the second column), and for Cronbach’s $\alpha$ and McDonald’s $\omega$ indexes (third column). The average amount of error used for generating the data is along the *x*-axis. In each panel of the first two columns, straight lines refer to the indexes computed by using the true values of the parameters, whereas the dashed lines to those estimated on the data. In the panels of the last column, straight lines refer to reliability estimated by using $\omega$ and dashed lines refer to reliability estimated by using $\alpha$.

Four main results arise. First, both KST-based indexes are monotonically decreasing in the amount of error in the data. This is true not only when they are estimated from the data (dashed lines) but also when they are computed by using the true parameter values (straight lines). Second, a systematic overestimation is observed for both indexes. The size of the overestimates is negligible in almost all conditions but it increases as both the amount of error in the data and the cardinality of true knowledge structure increase. Third, KS-Reliability index is systematically greater than RP-Reliability in both the estimated and the true values. This result could be due to the fact that RP-Reliability is based on the response patterns that contain noise, whereas KS-Reliability is based on the recovered knowledge states, that are what remains of the response patterns when noise is removed.

Concerning the results obtained for $\omega$ and $\alpha$ CTT-based reliability indexes (panels of the last column of Fig. 3), they perform very well in the unidimensional cases (rows 1 and 2), but their performances worsen a lot in the two multidimensional cases (rows 3 and 4). This result was somehow expected for both indexes.

**Fig. 4:**
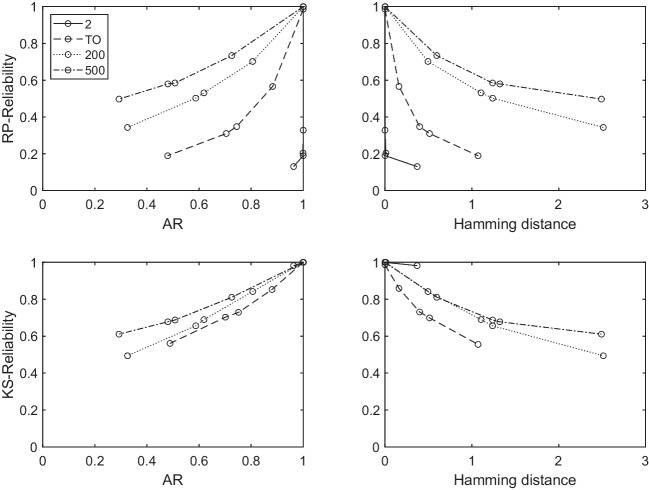
Results of the comparison between the two reliability indexes (*panels on the* *top* refer to the RP-Reliability, *panels on the* *right* to the KS-Reliability) with the AR (*panels on the left*) and the Hamming distance $K \Delta \hat{K}$ (*panels on the right*). Each panel displays the results of all conditions (see the legend for a reference)

Figure 4 shows the results of the comparison between the two reliability indexes (panels on the top refer to the RP-Reliability, panels at the bottom to the KS-Reliability) with the AR (panels on the left) and the Hamming distance $K \Delta \hat{K}$ (panels on the right). Each panel displays the results of all conditions (see the legend for a reference).

As expected, the relation between reliability indexes and the AR is monotonically increasing, whereas, the relation between reliability indexes and the Hamming distance is monotonically decreasing. This means that reliability increases as the proportion of subjects for which the true knowledge state is recovered increases and it decreases as the distance between the true and the estimated knowledge states increases. However, the shape of the relation between the assessment validity indexes (AR and Hamming distance) and the two reliability indexes seems to be different. In particular, the relation between RP-Reliability and both the assessment validity indexes (panels on the top) is clearly non-linear. This is true especially when $|\mathcal {K}|= 2$ and a total order is assumed on the item. On the other hand, the relation between KS-Reliability and both the assessment validity indexes (panels on the bottom) is more similar to a linear relation. This means that the KS-Reliability index could be informative about the AR. In real applications, the AR cannot be computed because the true knowledge state of a subject is unknown. Thus, the KS-Reliability index could be useful also in this direction.

## Empirical example

In this section, the proposed reliability indexes were applied to real data sets to provide evidence of their practical usability.

The data were obtained from the validation study of the Qualitative-Quantitative Evaluation of Depressive Symptomatology questionnaire (QuEDS; Spoto, Serra, Donadello, Granziol, & Vidotto, 2018). QuEDS investigates some symptoms of major depressive episode, through 41 items divided in three sub-scales: the Cognitive sub-scale (15 items), the Somatic sub-scale (14 items) and the Affective sub-scale (12 items). Each sub-scale was developed independently of the others. Each sub-scale was built using the *formal psychological assessment* methodology (Bottesi, Spoto, Freeston, Sanavio, & Vidotto, 2015; Spoto et al., 2010). The starting point for the construction of each sub-scale was a Boolean matrix containing items in rows and symptoms evaluated by those items in columns. The matrix was then used like a conjunctive skill map to define the structure and the clinical states (for further clinical and technical details see Spoto et al. 2018) For the present application, only Cognitive and Somatic sub-scales were used. The data were the same as in the validation study (the sample characteristics, the recruitment strategy, the structures and the validation data can be found in Spoto et al. 2018).

For the two Cognitive and Somatic subscales, Table 2 summarizes the test features in terms of the number of knowledge states in the structures (Column 2), the size of the validation sample (Column 3), mean values of $\hat{\beta }_q$ and $\hat{\eta }_q$ parameters (respectively Columns 4 and 5) estimated on the data, the average values $1-(\beta _q +\eta _q)$ computed across all the items belonging to each domain (Column 6), and the RP-Reliability and KS-Reliability (Columns 7 and 8).

**Table 2:** Results of the empirical application

Domain	$|\mathcal {K}|$	*N*	$\hat{\beta }_q$ (*sd*)	$\hat{\eta }_q$ (*sd*)	$1-\hat{\beta }_q-\hat{\eta }_q$	RP-Rel.	KS-Rel.
Cognitive	612	302	.16 (.20)	.02 (.05)	.82	.63	.91
Somatic	384	302	.07 (.08)	.02 (.03)	.91	.71	.96

For each subscale, the number of knowledge states in the structures is reported in Columns 2, the size of the validation sample is reported in Column 3, mean values (standard deviations) of $\hat{\beta }_q$ and $\hat{\eta }_q$ parameters estimated on the data are displayed in Columns 4 and 5, respectively, the average values $1-(\beta _q +\eta _q)$ computed across all the items belonging to each domain are in Column 6, and the RP-Reliability and KS-Reliability are in Columns 7 and 8

It can be observed that KS-Reliability is greater than or equal to .91 for both sub-scales. As shown by the simulations (Fig. 4), such values for KS-Reliability index suggest that: (i) the AR is also likewise high; and (ii) the average Hamming distance $d(K,\hat{K})$ is very close to zero. Moreover, RP-Reliability (which is always greater than .60) is always smaller or equal to KS-Reliability. Comparing the results obtained for the new RP-Reliability and KS-Reliability indexes with that obtained for the “item reliability” (Column 6), some interesting considerations arise. First, the “item reliability” is always smaller than or equal to KS-Reliability. This could be due to the fact that the “item reliability” computed by the formula $1-(\beta _q +\eta _q)$ does not take into account that also the probability of the knowledge states affects the overall reliability of the response patterns. In this specific application, the item reliability happens to be lower than KS-Reliability, but, in general, the inequality between their sizes could be also reversed. On the other hand, the “item reliability” is always higher than RP-Reliability. Finally, the absolute difference between “item reliability” and KS-Reliability is very low and it is smaller than the absolute difference between “item reliability” and RP-Reliability.

The results obtained in this application have an illustrative purpose only. In fact, it is worth noting that the sample size of the proposed empirical application is rather small, compared to the size of the knowledge structure. This may lead to rather high standard errors of the parameter estimates, affecting, in turn, the standard error of the estimate of the reliability indexes.

## Discussion and final remarks

The present article aimed at filling the gap in KST literature about a global reliability index. In fact, even if KST is nowadays among the most recent and grounded measurement approaches, differently from other measurement approaches like CTT, IRT, and even CDMs, no overall indexes have been developed in KST to estimate the reliability of the measure. In fact, in KST, reliability is either indirectly evaluated for each single item through the estimation of $\eta _q$ and $\beta _q$ error parameters, or evaluated for the adaptive assessment by means of the extra question procedures.

In this article, we first assessed the possibility of applying in KST the existing CTT methods for the estimation of reliability. We then verified that this cannot be done due to the fact that some fundamental assumptions of CTT conflict with KST by making the available indexes not suitable for this latter approach. We then proposed two new indexes for the assessment of reliability, based on the concepts of entropy and conditional entropy. The RP-Reliability index is used to obtain the reliability of the response pattern given the knowledge state, while the KS-Reliability even more importantly, refers to the reliability of the estimated knowledge of an individual. Some theoretical considerations as well as simulations and an empirical example on real data are provided within a study of the behavior of these indexes under a certain number of different conditions.

Simulation results suggest that (i) the two KST-based reliability indexes tend (as expected) to decrease as functions of the amount of error in the data; (ii) interestingly enough, the size of the KS-Reliability index tends to be greater than the RP-Reliability index; (iii) finally, CTT traditional indexes applied to data generated according to KST seem to provide an accurate estimation only in the unidimensional situation (i.e., the “empty-total” structure, and the linear order structure). The empirical example, on the one hand, highlighted the actual opportunity to use these indexes in real settings and, on the other hand, showed a possible argument in favor of the application of the two new indexes rather than the “item reliability” index used so far in concrete KST applications.

On the whole, the obtained results seem to indicate an overall adequate performance of the indexes as global indicators of reliability of the response patterns and of the estimated knowledge states. In fact, the observed performances are in line with what expected for a reliability index in the different conditions controlled in the simulations. Therefore, it seems reasonable to use these indexes together with all the fit indexes of the BLIM to test, on the one hand, the reliability of the model and, on the other hand, the reliability of the conducted assessment. For the latter, in particular, the KS-Reliability index allows to estimate the reliability of the assessment net of the random noise observed in the pattern. This issue is confirmed by the observation that, in both the simulation study and the empirical examples, the value of the KS-Reliability index is higher than that of the RP-Reliability index.

The two reliability indexes proposed in this article rely upon the assumption that complete data are available in an assessment (i.e., all the observed response patterns are subsets of the power set of *Q*). Therefore, they could be inappropriate as reliability indexes in the case of missing data. A proper adjustment of these indexes to this case is an issue that deserves further attention in future studies. This appears to be particularly important in KST. In fact, adaptive assessment, which naturally gives rise to missing data, is a common practice in this theory.

The problem of the evaluation of the reliability, is shared also by another approach which presents several similarities with KST, namely the Cognitive Diagnostic Models (CDM; Tatsuoka, 1985). In this framework, the focus is on the assessment of the attributes possessed by an individual who answers a set of items, rather than, as in KST, on the collection of items solved. Accordingly, the evaluation of reliability is shifted from the items to the underlying attributes. Therefore, although a number of correspondences have been shown between KST and CDM (Heller, Stefanutti, Anselmi, & Robusto, 2015), none of the indexes proposed in the latter for evaluating the reliability can be directly applied to the former. Future research should investigate in detail this issue.

Future research perspectives on this topic are open regarding (i) a deeper evaluation of the relationships between KS-Reliability and RP-Reliability indexes; (ii) how the probability distribution on the knowledge states affects the reliability of the model and of the assessment; (iii) the comparison between the performance in terms of reliability of the adaptive and standard assessment; (iv) the effect of the unidentifiability of some BLIM’s parameters on the estimated reliability indexes.

### Supplementary Information

Below is the link to the electronic supplementary material.

Supplementary file 1 (pdf 128 KB)

## Data Availability

All the data used in the empirical application can be found at the following OSF folder: https://osf.io/4dctj/?view_only=a5539bf65fd246a58cebe790a2981c59

## Code Availability

The MATLAB code to estimate the reliability indexes can be found at the following OSF folder: https://osf.io/4dctj/?view_only=a5539bf65fd246a58cebe790a2981c59

## References

[CR1] Anselmi, P., Stefanutti, L., de Chiusole, D., & Robusto, E. (2017). The assessment of knowledge and learning in competence spaces: The gain–loss model for dependent skills. *British Journal of Mathematical and Statistical Psychology,**70*(3), 457–479. 28211048 10.1111/bmsp.12095

[CR2] Bottesi, G., Spoto, A., Freeston, M. H., Sanavio, E., & Vidotto, G. (2015). Beyond the score: Clinical evaluation through formal psychological assessment. *Journal of personality assessment,**97*(3), 252–260. 25257993 10.1080/00223891.2014.958846

[CR3] Boyle, G. J. (1991). Does item homogeneity indicate internal consistency or item redundancy in psychometric scales? *Personality and individual differences,**12*(3), 291–294. 10.1016/0191-8869(91)90115-R

[CR4] Brown, W. (1910). Some experimental results in the correlation of mental abilities 1. *British Journal of Psychology, 1904-1920,**3*(3), 296–322. 10.1111/j.2044-8295.1910.tb00207.x

[CR5] Cover, T. M. (1999). *Elements of information theory*. John Wiley & Sons.

[CR6] Cronbach, L. J. (1951). Coefficient alpha and the internal structure of tests. *Psychometrika,**16*(3), 297–334. 10.1007/BF02310555

[CR7] de Chiusole, D., Stefanutti, L., Anselmi, P., & Robusto, E. (2015). Modeling missing data in knowledge space theory. *Psychological Methods,**20*(4), 506. 26651988 10.1037/met0000050

[CR8] Doignon, J.-P., & Falmagne, J.-C. (1985). Spaces for the assessment of knowledge. *International journal of man-machine studies,**23*(2), 175–196. 10.1016/S0020-7373(85)80031-6

[CR9] Doignon, J.-P., & Falmagne, J.-C. (1999). *Knowledge spaces*. Berlin - Heidelberg: Springer - Verlag.

[CR10] Dunn, T. J., Baguley, T., & Brunsden, V. (2014). From alpha to omega: A practical solution to the pervasive problem of internal consistency estimation. *British journal of psychology,**105*(3), 399–412. 24844115 10.1111/bjop.12046

[CR11] Falmagne, J.-C., Albert, D., Doble, C., Eppstein, D., & Hu, X. (2013). Knowledge spaces: Applications in education. Springer Science & Business Media.

[CR12] Falmagne, J.-C., & Doignon, J.-P. (1988). A class of stochastic procedures for the assessment of knowledge. *British Journal of Mathematical and Statistical Psychology,**41*, 1–23. 10.1111/j.2044-8317.1988.tb00884.x

[CR13] Falmagne, J. C., & Doignon, J. P. (1988). A Markovian procedure for assessing the state of a system. *Journal of Mathematical Psychology,**32*, 232–258. 10.1016/0022-2496(88)90011-9

[CR14] Falmagne, J.-C., & Doignon, J.-P. (2010). Learning spaces: Interdisciplinary applied mathematics. Springer Science & Business Media.

[CR15] Gulliksen, H. (2013). *Theory of mental tests*. Routledge.

[CR16] Hambleton, R. K., Swaminathan, H., & Rogers, H. J. (1991). Fundamentals of item response theory (Vol. 2). Sage.

[CR17] Heller, J. (2017). Identifiability in probabilistic knowledge structures. *Journal of Mathematical Psychology,**77*, 46–57. 10.1016/j.jmp.2016.07.008

[CR18] Heller, J., Stefanutti, L., Anselmi, P., & Robusto, E. (2015). On the link between cognitive diagnostic models and knowledge space theory. *Psychometrika,**80*(4), 995–1019. 25838246 10.1007/s11336-015-9457-x

[CR19] Lord, F. M. (1980). *Applications of item response theory to practical testing problems*. Routledge.

[CR20] Lord, F. M., & Novick, M. R. (1968). Statistical theories of mental test scores. Reading: Addison-Wesley.

[CR21] McDonald, R. (1999). Test theory: A unified treatment (1st ed.). Psychology Press. 10.4324/9781410601087

[CR22] Miller, M. B. (1995). Coefficient alpha: A basic introduction from the perspectives of classical test theory and structural equation modeling. *Structural Equation Modeling: A Multidisciplinary Journal,**2*(3), 255–273. 10.1080/10705519509540013 10.1080/10705519509540013

[CR23] Novick, M. R. (1965). The axioms and principal results of classical test theory. *ETS Research Report Series,**1965*(1), 1–18.

[CR24] Rasch, G. (1960). *Studies in mathematical psychology: I. Probabilistic models for some intelligence and attainment tests*. Oxford, England: Nielsen & Lydiche.

[CR25] Raykov, T. (1997). Scale reliability, cronbach’s coefficient alpha, and violations of essential tau-equivalence with fixed congeneric components. *Multivariate behavioral research,**32*(4), 329–353. 10.1207/s15327906mbr3204_2 26777071

[CR26] Schrepp, M. (2020). On the usage of cronbach’s alpha to measure reliability of ux scales. *Journal of Usability Studies, 15*(4).

[CR27] Shannon, C. E. (1948). A mathematical theory of communication. *The Bell system technical journal,**27*(3), 379–423. 10.1002/j.1538-7305.1948.tb01338.x

[CR28] Shevlin, M., Miles, J., Davies, M., & Walker, S. (2000). Coefficient alpha: a useful indicator of reliability? *Personality and individual differences,**28*(2), 229–237. 10.1016/S0191-8869(99)00093-8

[CR29] Sijtsma, K. (2009). On the use, the misuse, and the very limited usefulness of cronbach’s alpha. *Psychometrika,**74*(1), 107. 20037639 10.1007/s11336-008-9101-0 PMC2792363

[CR30] Spearman, C. (1910). Correlation calculated from faulty data. *British Journal of Psychology, 1904-1920,**3*(3), 271–295. 10.1111/j.2044-8295.1910.tb00206.x

[CR31] Spoto, A., Serra, F., Donadello, I., Granziol, U., & Vidotto, G. (2018). New perspectives in the adaptive assessment of depression: the ats-pd version of the queds. *Frontiers in Psychology,**9*, 1101. 30034352 10.3389/fpsyg.2018.01101 PMC6043690

[CR32] Spoto, A., Stefanutti, L., & Vidotto, G. (2010). Knowledge space theory, formal concept analysis, and computerized psychological assessment. *Behavior Research Methods,**42*(1), 342–350. 10.3758/BRM.42.1.342 20160314

[CR33] Spoto, A., Stefanutti, L., & Vidotto, G. (2012). On the unidentifiability of a certain class of skill multi map based probabilistic knowledge structures. *Journal of Mathematical Psychology,**56*(4), 248–255.

[CR34] Spoto, A., Stefanutti, L., & Vidotto, G. (2013). Considerations about the identification of forward-and backward-graded knowledge structures. *Journal of Mathematical Psychology,**57*(5), 249–254. 10.1016/j.jmp.2013.09.002

[CR35] Stefanutti, L., de Chiusole, D., Anselmi, P., & Spoto, A. (2020). Extending the basic local independence model to polytomous data. *Psychometrika,**85*, 684–715. 10.1007/s11336-020-09722-5 PMC7599199 32959202

[CR36] Stefanutti, L., de Chiusole, D., Gondan, M., & Maurer, A. (2020). Modeling misconceptions in knowledge space theory. *Journal of Mathematical Psychology,**99*, 102435. 10.1016/j.jmp.2020.102435

[CR37] Stefanutti, L., Heller, J., Anselmi, P., & Robusto, E. (2012). Assessing the local identifiability of probabilistic knowledge structures. *Behavior Research Methods,**44*(4), 1197–1211. 22588988 10.3758/s13428-012-0187-z

[CR38] Stefanutti, L., & Robusto, E. (2009). Recovering a probabilistic knowledge structure by constraining its parameter space. *Psychometrika,**74*(1), 83–96. 10.1007/s11336-008-9095-7

[CR39] Stefanutti, L., & Spoto, A. (2020). Blim’s identifiability and parameter invariance under backward and forward transformations. *Journal of Mathematical Psychology,**95*, 102314.

[CR40] Stefanutti, L., Spoto, A., & Vidotto, G. (2018). Detecting and explaining blim’s unidentifiability: Forward and backward parameter transformation groups. *Journal of Mathematical Psychology,**82*, 38–51.

[CR41] Tatsuoka, K. K. (1985). A probabilistic model for diagnosing misconceptions by the pattern classification approach. *Journal of Educational Statistics,**10*(1), 55–73. 10.3102/10769986010001055

[CR42] Vincent, S. B. (1912). The functions of the vibrissae in the behavior of the white rat... (Vol. 1) (No. 5). University of Chicago.

