# Supplementary material for: Reliability of a probabilistic knowledge structure
Source: Behav Res Methods. 2024 Jul 25;56(7):8022–37. doi: 10.3758/s13428-024-02468-3 (PMC11362261; doi:10.3758/s13428-024-02468-3)
Supplement: Supplementary file 1 — (pdf 128 KB) [file 13428_2024_2468_MOESM1_ESM.pdf]

Web Supplementary Material of the article "Reliability of a probabilistic knowledge structure"

Figure 1 displays the results obtained for Scenario 2 in the simulation study described in Section 3.1. The structure used for generating the data was $\mathcal{K}_{rand}$. Row panels represent the four considered types of error, whereas column panels represent the three amounts of error. In each panel, the true score t is along the x-axis, whereas the error term $\bar{\Delta}_t$ is along the y-axis. Error bars refer to standard deviations and circles in each panel indicate the expected amount of error ϵ.

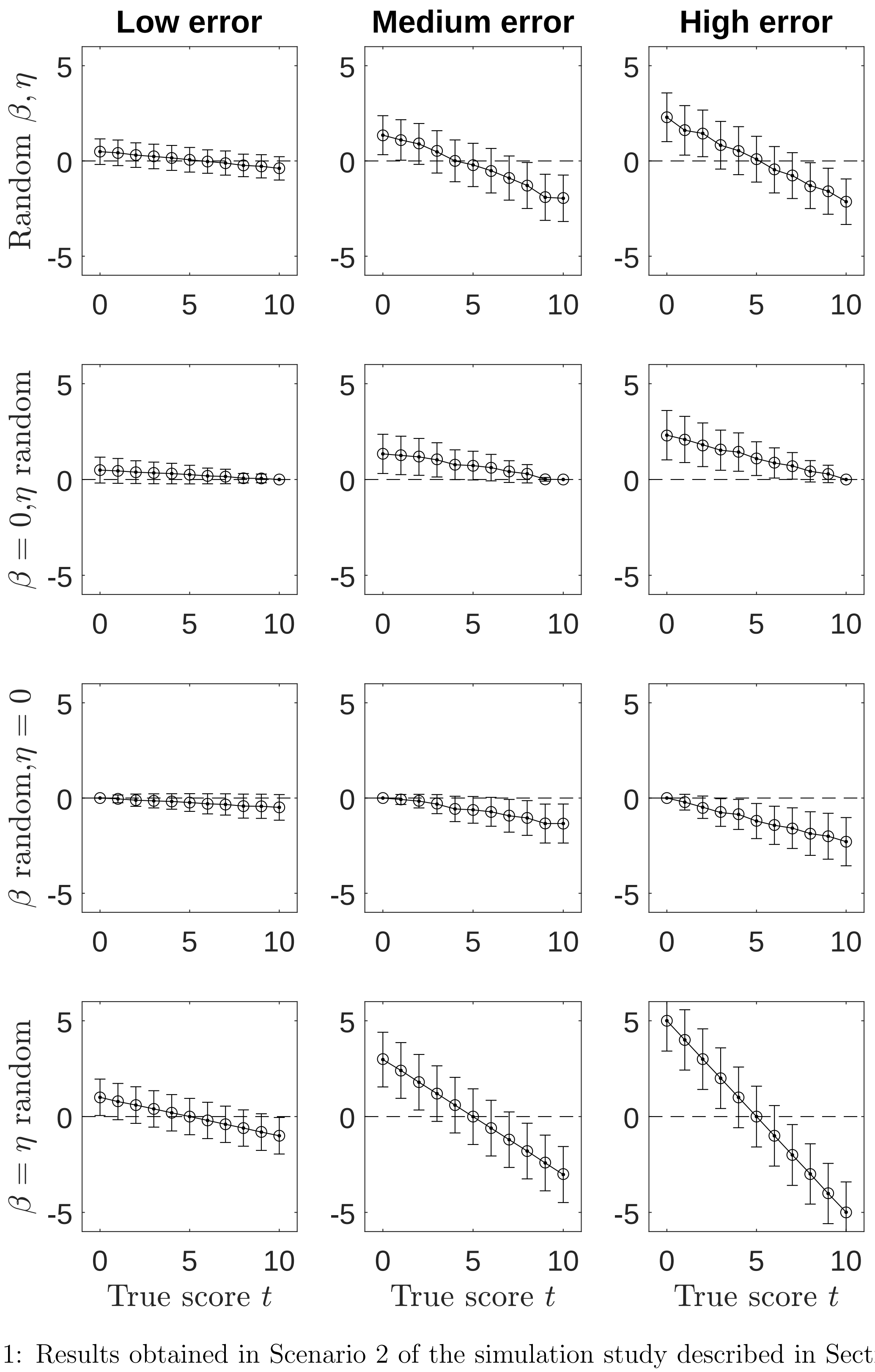

Figure 1: Results obtained in Scenario 2 of the simulation study described in Section 3.1. Error term $\bar{\Delta}_t$ as a function of the true score $t \in \{0, 1, \cdots, 10\}$. Error bars are standard deviations.
